# Analysis of Onboard Verification Flight Test for the Salinity Satellite Scatterometer

**DOI:** 10.3390/s23218846

**Published:** 2023-10-31

**Authors:** Yongqing Liu, Te Wang, Risheng Yun, Peng Liu, Wenming Lin, Di Zhu, Hao Liu, Xiangkun Zhang

**Affiliations:** 1 Key Lab of Microwave Remote Sensing, National Space Science Center, Chinese Academy of Sciences, Beijing 100190, China; liuyongqing20@mails.ucas.ac.cn (Y.L.);; 2 School of Electronic, Electrical and Communication Engineering, University of Chinese Academy of Sciences, Beijing 100049, China; 3 School of Marine Sciences, Nanjing University of Information Science and Technology, Nanjing 211544, China

**Keywords:** salinity satellite scatterometer, onboard verification flight, backscatter coefficient measurement

## Abstract

The upcoming Salinity Satellite, scheduled for launch in 2024, will feature the world’s first phased array radar scatterometer. To validate its capability in measuring ocean surface backscatter coefficients, this paper conducts an in-depth analysis of the onboard verification flight test for the Salinity Satellite scatterometer. This paper provides a detailed introduction to the system design of the Salinity Satellite scatterometer, which utilizes phased array radar technology and digital beamforming techniques to achieve accurate measurements of sea surface scattering characteristics. The paper elaborates on the derivation of backscatter coefficients, system calibration, and phase amplitude correction for the phased array scatterometer. Furthermore, it describes the process of the onboard calibration flight test. By analyzing internal noise signals, onboard calibration signals, and external noise signals, the stability and reliability of the scatterometer system are validated. The experiment covers both land and ocean observations, with a particular focus on complex sea surface conditions in nearshore areas. Through the precise analysis of backscatter coefficients, the paper successfully distinguishes the different backscatter coefficient characteristics between ocean and land. The research results effectively demonstrate the feasibility of the Salinity Satellite scatterometer for measuring backscatter coefficients in a phased array configuration, as well as its outstanding performance in complex marine environments.

## 1. Introduction

The ocean, as one of the most crucial natural resources on Earth, holds paramount significance in understanding climate change, marine environments, and ecosystems. Radar remote sensing technology serves as an efficient means for ocean observation, offering the capability to provide oceanic information on a global scale. However, due to the complexity and variability of the ocean surface, accurately studying sea surface scattering characteristics has always been one of the challenges in marine remote sensing research.

Global ocean salinity distribution and its intra-annual and interannual variations play a crucial role within the marine system, providing essential insights into global precipitation, evaporation, and water cycle changes. Sea surface salinity serves as a key parameter for monitoring and simulating global ocean circulation patterns and acts as a significant indicator of climate change. The Salinity Satellite is equipped with a combined active and passive salinity sensor, which includes a microwave scatterometer and a microwave radiometer [1]. Specifically, the microwave radiometer operates in the L-band frequency, which is highly sensitive to sea surface salinity variations [2,3]. However, the roughness of the sea surface can impact the precision of salinity measurements by the microwave radiometer. To mitigate the influence of surface roughness on radiometer measurement accuracy, co-frequency observations with the microwave scatterometer are necessary [4,5]. Through the observations provided by the microwave scatterometer, information about sea surface roughness can be obtained [6,7]. This information is subsequently used to calibrate the measurement results of the radiometer, thereby enhancing the accuracy and precision of salinity measurements [8,9,10].

The upcoming Salinity Satellite, expected to be launched in 2024, will carry the world’s first spaceborne microwave scatterometer using a phased array digital beamforming system. Phased array technology represents an advanced radar technique that employs the manipulation of phase and amplitude across multiple antenna elements to electronically steer and focus a beam of signals. This enables rapid and precise directional targeting and signal concentration. In comparison to traditional mechanical scanning systems, phased array technology offers greater flexibility and responsiveness, allowing for swift adaptation to varying observation requirements and achieving more accurate target localization and imaging [11,12,13].

**Figure 1:**
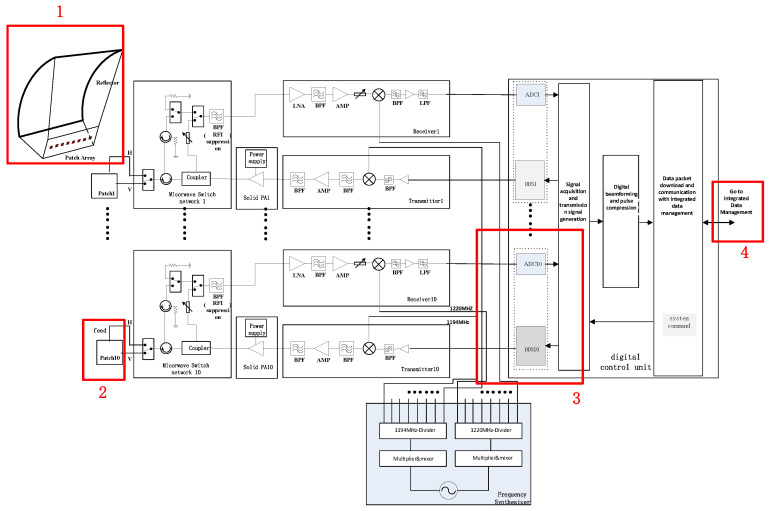
Schematic diagram of the onboard scatterometer system architecture.

**Figure 2:**
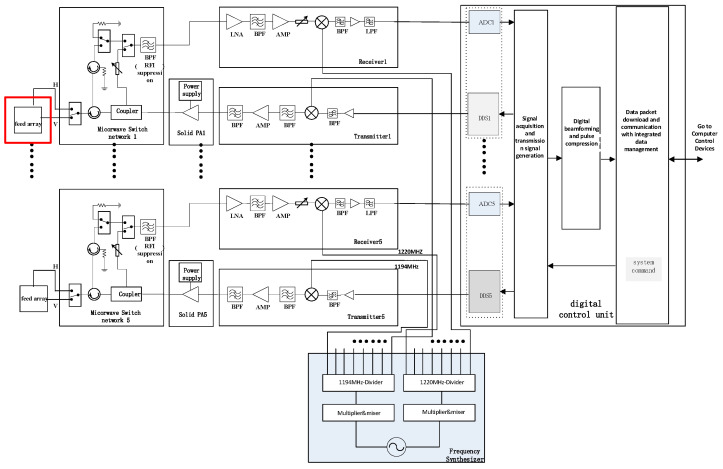
Schematic diagram of the airborne scatterometer system.

In the described phased array digital beamforming scatterometer system, the spaceborne system architecture consists of a shared reflector antenna, feed source, microwave front-end unit, power amplifier unit, transmitter unit, receiver unit, frequency synthesizer unit, and digital signal processing unit [14,15,16]. The array comprises ten transceiver channels, each operating independently. The system diagram is depicted in Figure 1. Given the size of the aircraft and the flight altitude for the flight tests, the system used in this testing is not identical to the actual spaceborne system. Instead, a portion of the spaceborne system equipment was employed. The structure of the airborne verification flight system is illustrated in Figure 2. There are some differences in the design and implementation of spaceborne and airborne scatterometer systems. These differences primarily involve the antenna, TR components (including the transmitter unit (T) and receiver unit (R)), the number of front ends, and data storage systems, highlighted in red boxes in Figure 1. Due to constraints in spacecraft space and power, the number of TR components, front ends, and antennas in the airborne scatterometer system is half that of the spaceborne scatterometer. Nevertheless, by adjusting the phase and amplitude of the array channel signals, digital beamforming (DBF) can still be achieved to test the system’s ability to precisely direct and focus signals. In contrast to the data storage of the spaceborne scatterometer, the airborne scatterometer system stores data on a computer. However, this does not impact the validation of the spaceborne scatterometer’s data acquisition and storage chain functionality. Another significant difference is that the scatterometer system used in aerial flight tests does not include a cylindrical reflector antenna; instead, an antenna array is used to replace the beam focusing function. In summary, while there are differences between the airborne and spaceborne scatterometer systems, the effective validation of critical functions, processes, and algorithms can still be achieved through a simplified system design. This facilitates the validation and optimization of system performance in more complex real-world environments and provides guidance for future enhancements.

The digital processing unit, serving as the core control unit of the Salinity Satellite scatterometer system, is responsible for coordinating the operation of other subsystems. Functionally, the digital processing unit can be divided into a timing generator, a signal generator, and a signal processor. The timing generator produces various timing control signals for the scatterometer’s operation. The signal generator generates signals such as the linear frequency modulation pulses required for system transmission and real-time processing. The signal processor is responsible for tasks like signal acquisition, real-time data processing onboard, and data packaging and transmission, as well as communication with the comprehensive data management subsystem.

The Salinity Satellite scatterometer is expected to provide a broader coverage and higher spatial resolution than the CFOSAT rotating fan beam scatterometer, thereby offering richer and more accurate oceanographic data. The utilization of phased array technology equips the scatterometer to rapidly gather multidimensional information, including sea surface wind fields, wave heights, ocean surface roughness, and more [17]. This enhancement contributes robust support to scientific research and practical applications in fields such as oceanography, meteorology, and environmental monitoring.

## 2. Overview of the Instrument

The Salinity Satellite scatterometer is an L-band (center frequency of 1.25 GHz) phased array radar. The array antenna utilizes digital beamforming technology to scan in different azimuthal angles with vertical (V) and horizontal (H) polarizations separately. The radar system operates using four distinct polarization configurations: VV (vertical transmit, vertical receive), HH (horizontal transmit, horizontal receive), VH (vertical transmit, horizontal receive), and HV (horizontal transmit, vertical receive). These polarization configurations constitute the measurement modes employed by the radar. Table 1 summarizes the main characteristics of the onboard instrument parameters and provides an overview of the main characteristics of the instrument during airborne testing. These parameters have been determined through various trade-offs between the signal-to-noise ratio (SNR), bandwidth size, platform constraints, and performance requirements [3,18].

**Figure 3:**
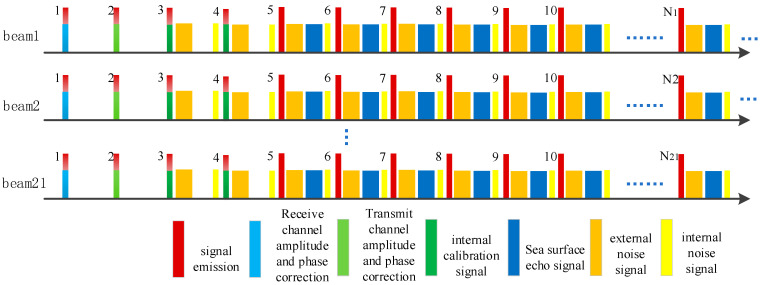
Pulse timing diagram.

Due to different operational scenarios, the pulse timing sequence during airborne operations differs from that during satellite operations. However, this does not affect the evaluation of the scatterometer system’s performance. The following explanation will focus solely on the timing sequence of the scatterometer when operating in orbit. In the nominal case, the Salinity Satellite scatterometer transmits vertically and horizontally polarized pulses alternatively. Table 2 presents the azimuth angle settings for each beam position of the Salinity Satellite scatterometer. The phased array antenna performs periodic scans at different azimuth angles, covering a total of 21 beam positions (−29° to 29°). The beam dwell time is 32 pulse repetition periods for each beam position, resulting in a complete azimuth scan cycle of 21 × 32 × 0.01 = 6.72 s. The first two pulses of each beam position are used for amplitude and phase calibration of the 10 transmitting and receiving channels. The third and fourth pulses of each beam position are primarily utilized for internal calibration, thermal noise measurement, or passive measurements similar to radiometric measurements. The subsequent 28 pulses of each beam position are employed for the VV, HH, VH, and HV backscatter measurement modes. During implementation, the number of transmitting and receiving pulses and the polarization configuration for each beam position can be flexibly adjusted using a timing lookup table. Figure 3 illustrates a schematic of the pulse transmission and reception sequence for a single beam position. Within a pulse cycle for a specific beam position, the sequence includes the timing for receiving channel amplitude and phase calibration and transmitting channel amplitude and phase calibration, as well as internal calibration, radiometric measurement, and noise measurement for HH and VV beams.

The transmitted pulse is written as (1):

(1) $$T\left(t\right)=\sqrt{E_{t}}P(t)e^{j2\pi \left(f_{c}+f_{dc}+\frac{1}{2}\mu t\right)t}$$

where *t* is the time from the onset of each pulse, $E_{t}$ is the total emitted energy, P(t) stands for the pulse power envelope such that $\int {P(t)}^{2}dt=1$, $f_{c}$ is the carrier frequency, $f_{dc}$ is the Doppler compensation frequency, and μ=1.5 MHz/0.95 ms is the chirp rate of the linear frequency-modulated pulse. In practice, the Doppler compensation frequency $f_{dc}$ is defined as a function of the beam azimuth angle. It can be configured for different channels using a look-up table (LUT) and is stored in a static random access memory (RAM) of the Salinity Satellite scatterometer data processing unit.

**Figure 4:**
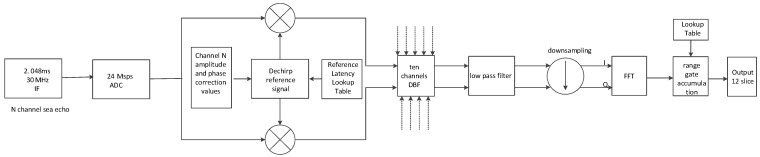
Signal processing flowchart.

During oceanic backscatter measurements, the microwave scatterometer operates alternately in HH, VV, HV, and VH polarizations, capturing the backscatter signals from the ocean surface under different polarizations. By adjusting the phase offsets of the phased array antenna elements, backscatter signals from different azimuth angles are acquired. These signals are then subjected to digital dechirping, coherent demodulation, and phase amplitude correction in each receiving channel of the phased array antenna. After the 30 MHz intermediate frequency (IF) signal undergoes undersampling by a 24 MHz sampling rate analog-to-digital converter (ADC), a 6 MHz signal is employed for coherent demodulation. By adjusting the phase offsets ($\phi_{i}$) for each array element appropriately, the direction of the synthesized beam pattern received can be controlled, enabling digital beamforming to steer the antenna’s beam pattern towards specific directions (The phase offset of the *i*-th antenna array element $\phi_{i}=\left(i-1\right)\frac{2\pi }{\lambda }d\sin\theta \sin\varphi$, where θ is the elevation observation angle of the antenna, and θ is the current azimuth angle of the antenna). After the synthesis of the 10 channel beams, the signal is filtered through an anti-aliasing filter and then downsampled by a factor of 12, to obtain baseband I/Q signals (orthogonal signals with a center frequency of 0 Hz). Following this, fast Fourier transform (FFT) processing is applied to the signal, and the squared magnitude is computed to derive the range distribution of the backscatter coefficient. The range distribution is then divided into distinct range bins based on a range bin splitting lookup table, which is used to calculate the energy values of the backscatter echoes in each range bin. This process allows for the determination of the energy distribution of backscatter signals at different distances from the radar. Since external noise is derived from the energy of external noise received by the receiving antenna, the data acquisition and processing mode for external noise signals is identical to that of the backscatter signals. Internal calibration data are utilized in the calculation of the backscatter coefficient to eliminate fluctuations in transmission power and receiving gain. This process allows for the accurate inversion of the target’s backscatter coefficient. Internal calibration signals are obtained by coupling out signals from each of the 10 transmit channels. These signals are then received and sampled by the receiving channels. The data acquisition and processing mode for internal noise signals is quite similar to that of the backscatter signals. The only difference is that the reference signal duration used for pulse compression processing is different. This process yields energy values for internal calibration. Internal noise signals originate from internal noise sources. The processing of internal noise signals is identical to the processing of internal calibration signals. This similarity allows for the determination of energy values for internal noise, following the same steps as those used for processing internal calibration data. Figure 4 illustrates the real-time processing flowchart for both the backscatter signals and the internal calibration signals.

In summary, the onboard processing measures four essential quantities: (1) the backscatter energy $E_{r,i}$ (*i* = 1, 2,…, 12) of 12 slices; (2) the external noise $E_{ne,i}$ (radiometric mode); (3) the internal noise $E_{ni,i}$ (matched impedance); and (4) the internal calibration energy $E_{c}$. The raw outputs of all the above channels are digital numbers rather than real energy value.

## 3. $\sigma_{0}$ Estimation

The slice measurement $E_{r,i}$ consists of thermal noise ($E_{n}$)and backscatter from Earth surface ($E_{s,i}$). To calculate the radar backscatter coefficient ($\sigma_{0}$), one needs to estimate the noise contribution (instrument plus Earth scene), subtract it from Er, and then relate the surface $\sigma_{0}$ to $E_{s,i}$ according to the radar equation. Therefore, the surface backscatter value of the *i*-th slice can be expressed by the following formula:

(2) $$E_{s,i}=E_{r,i}-C_{n}E_{ne,i}$$

where $C_{n}$ is a constant noise correction factor. It is important to note that $C_{n}$ is different for each antenna beam. Assuming that the backscatter coefficient $\sigma_{0}$ is constant for a particular slice, the received echo energy of the i-th slice by the antenna can be represented as:

(3) $$E_{s,i}^{a}=X_{i}*\sigma_{0}$$

where $X_{i}$ is the radar calibration factor, expressed as follows:

(4) $$X_{i}=\frac{P_{t}\lambda^{2}}{{\left(4\pi \right)}^{3}L}\sum_{i\in F}\frac{\Delta A_{i}G_{i}^{2}}{r_{i}^{4}}\sum_{k=k_{s}}^{k_{e}}{\left\{\frac{\sin\left[\pi N_{j}\left(f_{b,i}T-\frac{k}{N}\right)\right]}{\sin\left[\pi \left(f_{b,i}T-\frac{k}{N}\right)\right]}\right\}}^{2}$$

Here, the echo signal is modeled as the sum of echo scattering from a sufficient number of independent surface patches within the footprint F. Each patch has an area $\Delta A_{i}$ and is at a distance $r_{i}$ from the receiver.

The relevant parameters involved in Equation (4) include: transmit power $P_{t}$, wavelength λ, bidirectional antenna gain $G_{i}^{2}$, bidirectional transmission loss L, sampling period T, FFT length N, boundaries of the slice bandwidth indicated by indices $k_{s}$ and $k_{e}$, and the length of the echo from the *i*-th region captured by distance gates $N_{i}$. In the Equation, $f_{b,i}=f_{d,i}+f_{dc}+s\mu \left(t_{0}-t_{i}\right)$, where $f_{b,i}$ is the central frequency of the *i*-th FFT frequency point, $f_{d,i}$ is the Doppler frequency shift at the observation point, $f_{dc}$ is the Doppler compensation frequency, sμ is the signed frequency slope, $t_{0}$ and $t_{i}$ are reference delays and the round-trip microwave propagation time from the satellite to the observation point, respectively.

Internal calibration is, in fact, used to determine the ratio of the received signal to the transmitted power, $E_{s,i}^{a}/P_{t}$. As shown in the following equation:

(5) $$\frac{E_{s,i}^{a}}{P_{t}}=K_{s}\frac{E_{s,i}}{P_{0}^{c}}$$

where $K_{s}$ is an overall calibration constant that takes into account the attenuator of the internal calibration, as well as the directional couplers of the transmitter and receiver, and the loss factors associated with two directional couplers related to the internal calibration path. In the frequency domain, the equivalent output $P_{0}^{c}$ of the internal calibration chain is given by:

(6) $$P_{0}^{c}=\frac{N*E_{c}}{\tau_{p}}$$

In Equation (6), $\tau_{p}$ represents the pulse duration. Finally, the backscatter coefficient $\sigma_{0}$ can be expressed as:

(7) $$\sigma_{0}=K_{s}\frac{E_{s,i}*\tau_{p}}{N*E_{c}*X_{i}^{\prime }}$$

Here, $X_{i}^{\prime }$ is the same as (4) but excluding the transmitted power $P_{t}$.

## 4. System Calibration and Amplitude and Phase Correction

### 4.1. System Calibration

Accurate measurement of the backscatter coefficient requires system calibration [19]. A phased array scatterometer is an active remote sensing instrument used to measure the backscatter characteristics of targets. In practical applications, some factors in the system, such as jitter, temperature drift, etc., may cause the gain of the received signal or the transmitted signal to fluctuate, thus affecting the accuracy of the measurement results [20,21]. To mitigate the effects of channel-to-channel gain imbalances and cross-talk, and to obtain the true target scattering matrix, phased array scatterometers employ a relative calibration approach for system calibration [22,23,24]. During flight or observation, the phased array scatterometer switches to internal calibration mode. In this mode, a switching mechanism is activated to prevent the transmit signal from being radiated through the antenna. Instead, the signal is looped back to the receiving channel through the front end. Through this procedure, the phased array scatterometer can perform real-time correction for gain fluctuations during actual observations, thereby ensuring the precision of backscatter coefficient measurements [25].

The signal energy and noise energy measured in the internal calibration loop are represented by Equations (8) and (9), respectively:

(8) $$E_{r,cal}=L_{f}G_{re}P_{t}\tau_{p}+G_{re}B_{s}N_{o,cal}\tau_{s,g}\approx L_{f}G_{re}P_{t}\tau_{p}$$

(9) $$E_{n,cal}=L_{f}G_{m}P_{t}\tau_{p}+G_{m}B_{n}N_{o,cal}\tau_{n,g}\approx L_{f}G_{m}P_{t}\tau_{p}$$

In the equation, $L_{f}$ represents the total loss of the internal calibration loop’s coupler and attenuator. $P_{t}$ and $\tau_{p}$ are the peak power and pulse width of the transmitted pulse, respectively. $G_{re}$ and $G_{m}$ are the gains of the signal channel and noise channel filters, which can be treated as constants for the digital signal processing process. $B_{s}$ and $B_{n}$ are the bandwidths of the echo and noise channels, respectively. $\tau_{s,g}$ and $\tau_{n,g}$ are the durations of the echo receive window and the noise receive window, and for the Salinity Satellite scatterometer, the following relationship exists: $\tau_{s,g}=\tau_{n,g}$. $N_{o,cal}$ represents the noise power spectrum in the internal calibration mode. Due to the significantly higher energy of the internal calibration compared to the noise energy (>50 dB), the noise energy in the above two equations can be neglected. Therefore, through internal calibration, the ratio of the gain of the signal channel to the gain of the noise channel can be determined as β ($\beta =G_{re}/G_{m}$), and $G_{re}*P_{t}*\tau_{p}$ (or $G_{m}*P_{t}*\tau_{p}$). The ratio of the effective bandwidth of the noise channel to the signal channel can be defined as α and can be represented as follows:

(10) $$\alpha =\frac{1}{\beta }\frac{N_{s}}{N_{n}}$$

where $N_{s}=G_{re}B_{s}N_{o,cal}\tau_{s,g}$ represents the energy of the noise in the signal channel, and $N_{n}=G_{m}B_{n}N_{o,cal}\tau_{n,g}$ represents the energy of the noise in the noise channel. In this way, by determining the value of α, the energy of the echo can be determined.

In the backscatter coefficient measurement mode, the total energy of the received sea surface echo ($E_{r}$) and the total energy of the external noise ($E_{n}$) can be expressed as follows:

(11) $$E_{r}=G_{re}P_{s}\tau_{p}+G_{re}B_{s}N_{o}\tau_{s,g}$$

(12) $$E_{n}=G_{m}B_{n}N_{o}\tau_{n,g}$$

In the above formula, $P_{s}$ and $B_{s}$ respectively represent the total power and total bandwidth of the echo. The echo energy $E_{e}$ of the signal channel can be obtained from the Equations (8), (9), (11) and (12) as follows:

(13) $$E_{e}=G_{re}P_{s}\tau_{p}=\frac{E_{r}-\alpha \beta E_{n}}{1-\alpha }$$

### 4.2. Amplitude and Phase Correction

The Salinity Satellite scatterometer employs phased array architecture and utilizes digital beamforming technology to achieve cross-track scanning. However, in practical applications, during the process of digital beam synthesis, various factors, such as hardware performance parameters and environmental conditions among different channels, can affect the signals. Factors such as transmission path loss, device nonlinearity, and temperature environment may cause variations in signal amplitude and phase. These factors can result in inconsistent amplitude and phase among different channels, leading to distorted synthesized transmit pulse beams. This distortion can compromise the quality of digital beam synthesis and subsequently impact the accuracy of measurement results [26,27,28].

**Figure 5:**
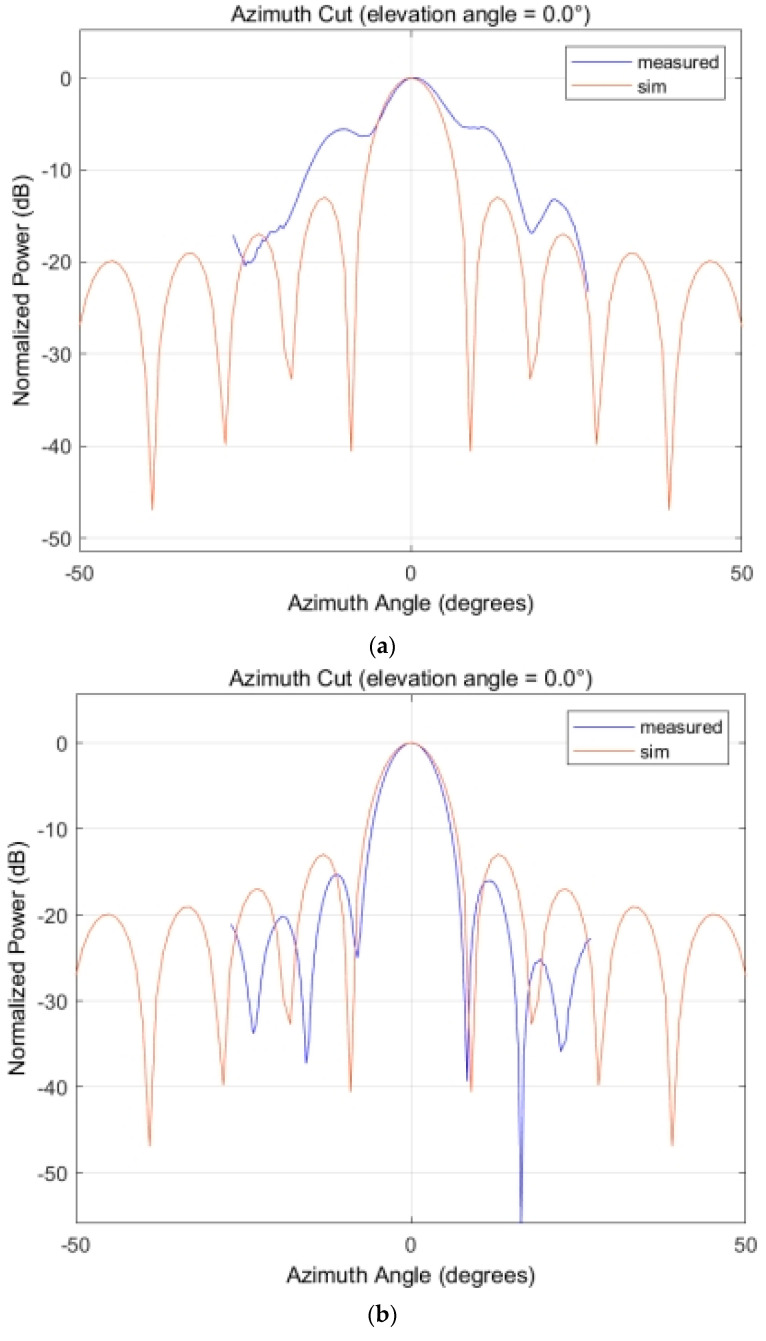
Amplitude and phase correction comparative analysis: (**a**) antenna pattern without amplitude and phase correction; (**b**) antenna pattern with amplitude and phase correction.

To address this issue, it is necessary to correct the amplitude and phase for each channel to ensure consistency among the signals, thereby improving the accuracy of backscatter coefficient measurements [29,30]. In this system, the inherent single-frequency signal transmitted by the system is used for the phase calibration and amplitude calculation for each channel. This process achieves amplitude and phase correction for each channel. The calibration results are depicted in Figure 5, where (a) shows the antenna pattern of the transmit pulse without amplitude and phase correction, and (b) shows the antenna pattern after correction. From the results, it can be observed that without amplitude and phase correction, the synthesized beam’s main lobe broadens, sidelobe levels increase, and beam distortion occurs. After correction, the quality of digitally synthesized beams aligns closely with theoretical simulations. Although the measured main lobe narrows and sidelobes decrease compared to theoretical simulations, this is likely due to the optimization measures taken during the implementation of the phase calibration algorithm. These optimizations, achieved through hardware resource allocation and parallel computation, effectively reduce amplitude and phase errors in the system. Consequently, the observed effect of narrowing the main lobe and reducing sidelobes is a positive outcome. It indicates that our calibration algorithm improves system performance without introducing additional errors or losses. On the contrary, by reducing the main lobe width and lowering sidelobe levels, the measurement accuracy of the system is enhanced.

## 5. Airborne Experimental Verification

**Figure 6:**
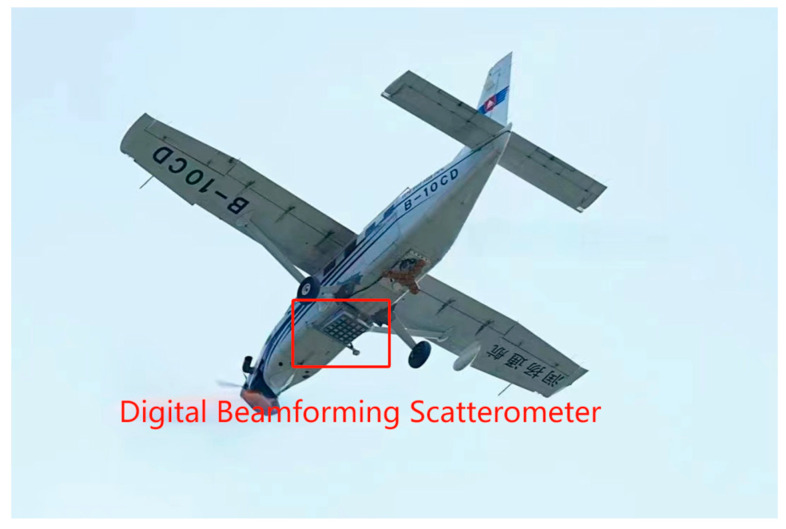
Airborne flight test platform.

To validate the feasibility of using the radar scatterometer system for measuring sea surface roughness, flight experiments were conducted by the phased array microwave scatterometer from 12 July to 14 August 2023, in Yantai City, Shandong Province. The experiments covered both land and ocean observations. The land observation area included the Yantai Port region, while the ocean observation area was located in the nearshore region. The flight platform for the airborne validation experiment is illustrated in Figure 6, with the digital beamforming scatterometer mounted on the underside of the aircraft. During the experiments, the airborne platform operated at an altitude of approximately 3 km with a speed of about 200 km/h. The sea surface conditions in the nearshore coastal area were more complex compared to the open ocean. For the microwave scatterometer, factors such as the frequent presence of vessels, potential small islands or reefs, and the variability of wind fields caused by interactions between the sea and land can affect the radar’s backscatter coefficient.

**Figure 7:**
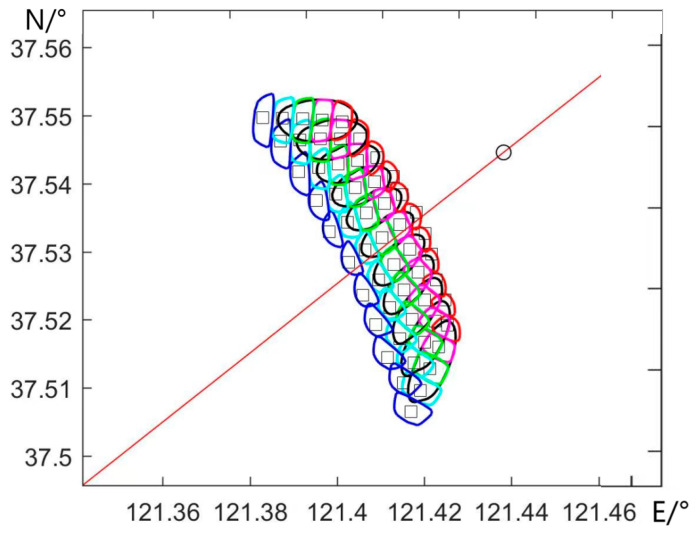
Schematic diagram of antenna footprint and slice division.

Figure 7 depicts a schematic diagram illustrating the airborne scatterometer’s beam scanning footprint and slice division. In the diagram, the black elliptical regions represent the antenna footprints of 11 beam positions. The colored blocks delineate the division of each footprint into slices, and each footprint can be further divided into five slices. This visual representation provides an overview of how the scatterometer’s beams cover the designated area and how these beams are subdivided into individual slices for analysis. The segmentation of each footprint into multiple slices allows for a detailed examination of backscatter characteristics within specific portions of the observed region.

In Level 1A products, three key parameters, essential for monitoring the performance of the phased array scatterometer, are recorded. These parameters encompass internal noise signal, external noise signal, and internal calibration signal. As mentioned earlier in this article, the internal noise signal is influenced by factors such as matched impedance and receiver temperature, while the external noise signal is subject to control by receiver and observation scene temperature. The internal calibration signal reflects variations in system gain and losses apart from antenna gain. Therefore, the assessment of the system’s operational status can be conducted through the analysis of internal noise signals, external noise signals, and internal calibration signals. This is because the stability of internal noise signals can reflect whether there is significant interference from system components within the scatterometer system. The intensity of external noise signals, on the other hand, indicates whether the external environment is experiencing extreme conditions. Internal calibration signals, in turn, provide insights into the stability of the system’s internal gain, enabling the detection of potential faults within the scatterometer system. These faults may include common issues such as array channel failures or feed source damage. Given that the channels within the phased array scatterometer array are independent of each other and follow a consistent implementation, this flight test experiment specifically focused on analyzing a single channel. This focused analysis facilitates a comprehensive assessment of the performance of individual channels, aiding in the identification of any discrepancies, deviations, or irregularities that might arise during operational scenarios.

The analysis of the results for each parameter signal is as follows.

**Figure 8:**
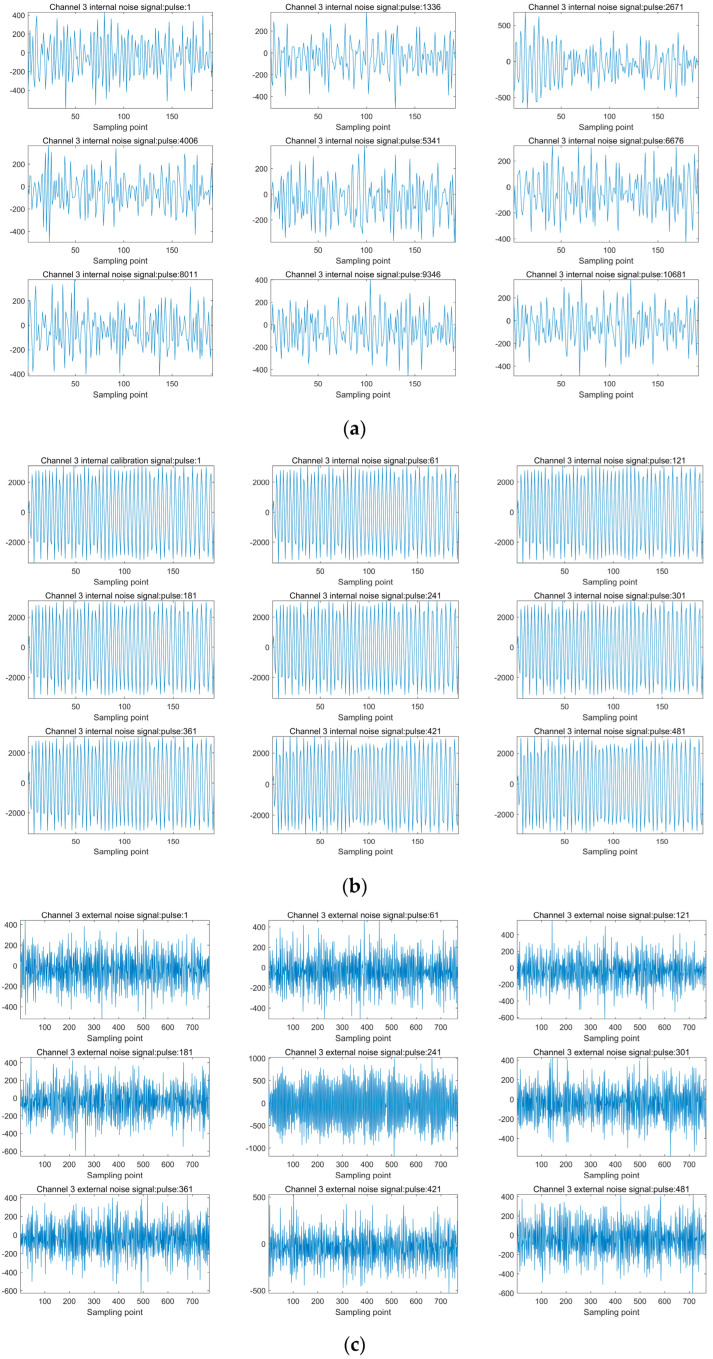
Scatterometer airborne test signal analysis: (**a**) channel three internal noise signal; (**b**) channel three internal calibration signal; (**c**) channel three external noise signal.

Figure 8a displays the variation of the internal noise signal for channel three over time. It can be observed that the values of the internal noise signal fluctuate within the range of −400 to 400, showing a relatively stable trend. This indicates that the internal noise signal experiences limited variation across a series of pulse moments, reflecting little change in matching impedance and receiver temperature during the measurement period.

Figure 8b illustrates the variation of the internal calibration signal for channel three. The waveform of the internal calibration chirp signal remains relatively stable at different pulse moments, with amplitude values ranging from −2500 to 2500. It is important to note that this amplitude value is significantly higher than that of the internal noise signal. This higher amplitude value implies a relatively high signal-to-noise ratio in the internal calibration mode, enabling accurate calibration and measurement of changes in system gain.

Figure 8c shows the variation of the external noise signal received by channel three at different pulse moments. The amplitude of the external noise signal fluctuates within the range of −600 to 600, exhibiting a relatively stable amplitude. This suggests that the external environment remained relatively stable during the measurement period, without the occurrence of sudden strong signal interference sources.

In conclusion, based on the analysis of the flight test data, it can be inferred that the Salinity Satellite scatterometer exhibited a fairly stable performance during the flight test period. These analysis results provide strong support for further assessing instrument performance, diagnosing potential issues, and verifying system stability.

**Figure 9:**
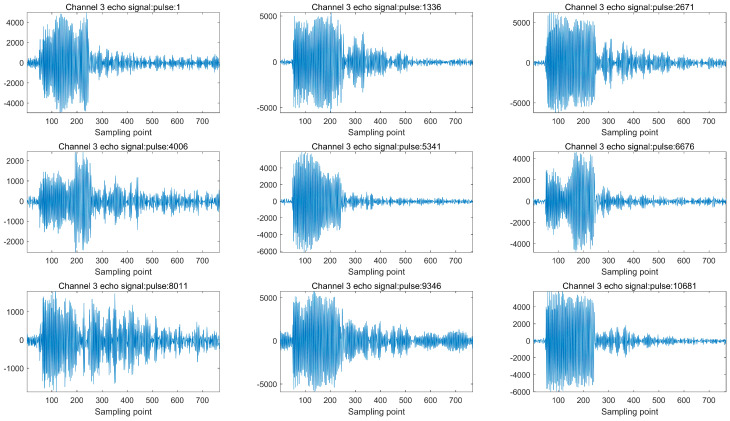
Channel three echo signal.

In the measurement mode of the scatterometer, the phased array microwave scatterometer scans and measures cyclically between different wave positions. During the airborne calibration flight test, the scanning time for each beam position is 17.6 milliseconds, and the system operates within a bandwidth of 2 MHz. In the ocean flight experiments conducted in the measurement mode, the acquired echo data are shown in Figure 9. Despite the relatively low signal-to-noise ratio and less noticeable signal variations across the channels, the consistency of the echo performance among the channels within the array can be determined based on the peak points of the signals.

**Figure 10:**
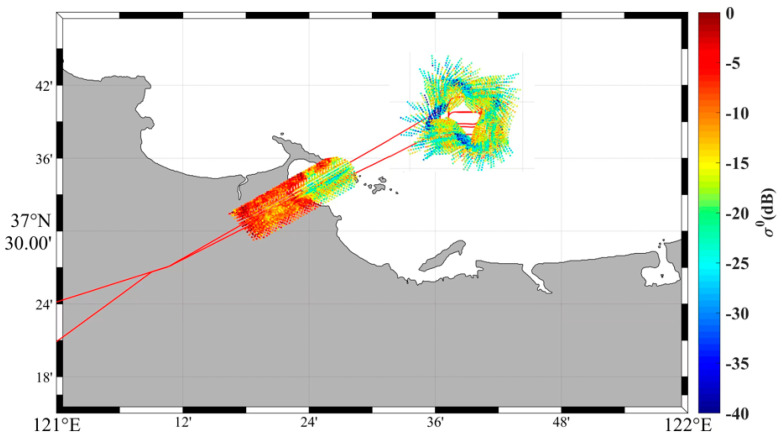
Distribution of backscatter coefficient on offshore platform.

Figure 10 presents the spatial distribution of backscatter coefficients measured in the airborne experiment of the digital beamforming scatterometer [31]. In the figure, the red trajectory represents the flight path of the test aircraft, while the data points represent the actual measured backscatter coefficient values. The upper part of Figure 10 displays the distribution obtained during the aircraft’s flight around the offshore platform. The measured backscatter coefficients align with the expected patterns, but some strong scattering points are observed. These may be influenced by the presence of ships navigating in the surveyed sea area, requiring further analysis in subsequent studies. The lower part of Figure 10 shows the distribution at the boundary between land and sea, where there is a clear distinction in the backscatter coefficients between the marine and terrestrial regions, creating a distinct demarcation line between the ocean and the land.

**Figure 11:**
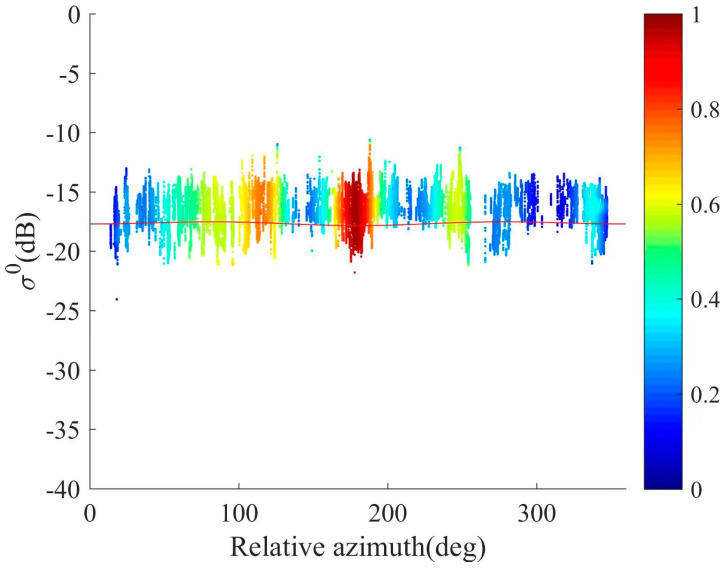
$\sigma_{0}$ azimuth Modulation and GMF model comparison.

Additionally, this study also compared and analyzed the azimuth modulation of the measured data from the airborne experiments of the Salinity Satellite scatterometer with the GMF (geophysical model function) model [32,33,34]. Figure 11 presents an illustrative plot of the variation in backscatter coefficients with the azimuth angle, for an incident angle of 38°. The red curve represents the conceptual variation of L-band backscatter coefficients with the azimuth angle, according to the GMF model. In contrast to the Ku-band, the modulation effect of L-band backscatter coefficients with the azimuth angle is less pronounced. The scatter points on the plot reflect the distribution of measured data obtained from the airborne experiment. Upon observing the data in the figure, it is evident that the results obtained by the Salinity Satellite scatterometer align closely with the changing trend predicted by the GMF model, albeit with minor discrepancies. This experiment provides initial evidence for the feasibility of using phased array scatterometers for measuring ocean backscatter coefficients. Going forward, efforts will be dedicated to further improving the performance of this scatterometer system to achieve higher measurement accuracy and reliability.

## 6. Conclusions

Through systematic experimental design and thorough data analysis, the aim of this study was to evaluate the feasibility of using the Salinity Satellite scatterometer as a phased array system for measuring backscatter coefficients in the ocean. In the experiments, we took into account the complex ocean observation environment, with a special focus on nearshore coastal areas. The results demonstrated that the Salinity Satellite scatterometer is capable of effectively distinguishing between land and sea boundaries. By monitoring key parameters such as internal noise signals, external noise signals, and internal calibration signals, we were able to assess the performance of the scatterometer, diagnose potential configuration changes, hardware faults, and instrument drift, showcasing its stability and reliability in challenging environments.

Therefore, based on the experimental data and analysis results from this study, we conclude that the Salinity Satellite scatterometer, operating as a phased array system for measuring backscatter coefficients, is a feasible method with potential practical applications. It provides robust technological support for marine remote sensing and environmental monitoring, contributing significantly to advancing ocean observation technology and deepening our understanding of ocean-atmosphere interaction mechanisms. Future research could expand the scope of experiments, optimize system design, and achieve even more precise backscatter coefficient measurements and ocean environmental monitoring.

## Figures and Tables

**Table 1:** List of the main parameters of spaceborne scatterometer.

Parameters	Values (Spaceborne)	Values (Airborne)
Center frequency	1.25 GHz	1.25 GHz
Bandwidth of transmitted signal	1.5 MHz	2 MHz
Peak power	200 W	100 W
Pulse repetition frequency (PRF)	100 Hz	6.667 KHz
Pulse width	0.95 ms	8 us
Bandwidth of received signal	3 MHz	3 MHz

**Table 2:** Antenna scan angles of Salinity Satellite scatterometer.

beam	1	2	3	4	5	6	7	8	9	10	11
Angle/°	−29	−26.8	−24.6	−21.8	−18.9	−15.9	−12.9	−9.9	−6.8	−3.8	0
beam	12	13	14	15	16	17	18	19	20	21	
Angle/°	3.8	6.8	9.9	12.9	15.9	18.9	21.8	24.6	26.8	29	

## Data Availability

The data presented in this study are available in this article.

## References

[1] Zhang X., Liu H. Design of phased array microwave scatterometer with digital beam forming technique in active and passive combining observation system for sea surface salinity. Proceedings of the 2013 IEEE International Geoscience and Remote Sensing Symposium—IGARSS.

[2] Freedman A., McWatters D., Spencer M. The Aquarius Scatterometer: An Active System for Measuring Surface Roughness for Sea-Surface Brightness Temperature Correction. Proceedings of the 2006 IEEE International Symposium on Geoscience and Remote Sensing.

[3] Spencer M., Wu C., Long D. (2000). Improved resolution backscatter measurements with the SeaWinds pencil-beam scatterometer. IEEE Trans. Geosci. Remote Sens..

[4] Reagan J., Boyer T., Antonov J., Zweng M. (2014). Comparison analysis between Aquarius sea surface salinity and World Ocean Database in situ analyzed sea surface salinity. J. Geophys. Res. Oceans.

[5] Martin A.C.H., Boutin J., Hauser D., Dinnat E.P. (2014). Active-passive synergy for interpreting ocean L-band emissivity: Results from the CAROLS airborne campaigns. J. Geophys. Res. Oceans.

[6] Ma W., Liu G., Yu Y., Du Y. (2021). Roughness correction method for salinity remote sensing using combined active/passive observations. Acta Oceanol. Sin..

[7] Hwang P.A., Ainsworth T.L. (2020). L-Band Ocean Surface Roughness. IEEE Trans. Geosci. Remote Sens..

[8] Wang N.-Y., Vesecky J., Fischer K., Onstott R., Shuchman R. Sea surface temperature estimate using active/passive microwave remote sensing: Outdoor experiment and model comparison. Proceedings of the 1995 International Geoscience and Remote Sensing Symposium, IGARSS ‘95. Quantitative Remote Sensing for Science and Applications.

[9] Hwang P.A. (2011). Foam and Roughness Effects on Passive Microwave Remote Sensing of the Ocean. IEEE Trans. Geosci. Remote Sens..

[10] Koblinsky C.J., Hildebrand P., LeVine D., Pellerano F., Chao Y., Wilson W., Yueh S., Lagerloef G. (2003). Sea surface salinity from space: Science goals and measurement approach. Radio Sci..

[11] Arnieri E., Boccia L., Amendola G., Glisic S., Mao C., Gao S., Rommel T., Penkala P., Krstic M., Yodprasit U. (2019). An Integrated Radar Tile for Digital Beamforming X-/Ka-Band Synthetic Aperture Radar Instruments. IEEE Trans. Microw. Theory Tech..

[12] Chen Z., Liu J., Li L. Design of scalable beam steering system of phased array radar. Proceedings of the 2011 IEEE CIE International Conference on Radar.

[13] Kinghorn T., Scott I., Totten E. Recent advances in airborne phased array radar systems. Proceedings of the 2016 IEEE International Symposium on Phased Array Systems and Technology (PAST).

[14] Garrod A. Digital modules for phased array radar. Proceedings of the International Symposium on Phased Array Systems and Technology.

[15] Herd J.S., Conway M.D. (2015). The Evolution to Modern Phased Array Architectures. Proc. IEEE.

[16] Fenn A.J., Temme D.H., Delaney W.P., Courtney W.E. (2000). The development of phased array radar technology. Lincoln Lab. J..

[17] Groger I., Sander W., Wirth W.-D. (1990). Experimental phased array radar ELRA with extended flexibility. IEEE Aerosp. Electron. Syst. Mag..

[18] Liu J., Lin W., Dong X., Lang S., Yun R., Zhu D., Zhang K., Sun C., Mu B., Ma J. (2020). First Results from the Rotating Fan Beam Scatterometer Onboard CFOSAT. IEEE Trans. Geosci. Remote Sens..

[19] Spencer M.W., Tsai W.-Y., Institute of ELECTRIC and Electronic Engineer NASA Scatterometer (NSCAT) pre-launch calibration results and post-launch calibration plan. Proceedings of the 1995 International Geoscience and Remote Sensing Symposium, IGARSS ‘95. Quantitative Remote Sensing for Science and Applications.

[20] Juha H., Hallikainen M.T., Makynen M. (1999). Calibration accuracy of the HUTSCAT airborne scatterometer. IEEE Trans. Geosci. Remote Sens..

[21] Tsai W.Y., Graf J.E., Winn C., Huddleston J.N., Dunbar S., Freilich M.H., Wentz F.J., Long D.G., Jones W.L. (1999). Postlaunch sensor verification and calibration of the NASA scatterometer. IEEE Trans. Geosci. Remote Sens..

[22] Rostan F., Ulrich D., Schied E., Heer C., Østergaard A. In-Flight Calibration of The Metop-Sg Sca Wind Scatterometer. Proceedings of the IGARSS 2018—2018 IEEE International Geoscience and Remote Sensing Symposium.

[23] Alsabah R., Al-Sabbagh A., Zec J. Calibration of RapidScat scatterometer. Proceedings of the 2017 IEEE Microwaves, Radar and Remote Sensing Symposium (MRRS).

[24] Yoho P., Anderson A., Long D., Lux J., Adams J., Cheng F. SeaWinds on QuikSCAT calibration using a calibration ground station. Proceedings of the IGARSS IEEE 2000 International Geoscience and Remote Sensing Symposium. Taking the Pulse of the Planet: The Role of Remote Sensing in Managing the Environment.

[25] Ailing L., Xiaoning W., Axin J., Lixia L. Calibration/validation of spaceborne microwave scatterometer on HY-2A. Proceedings of the 2014 IEEE International Geoscience and Remote Sensing Symposium (IGARSS 2014).

[26] Jin X., Xu D., Wang H. A Method of Channel Calibration Using Periodic Monitoring for Phased Array Radar Based On Fpga. Proceedings of the IET International Radar Conference: IET IRC 2020.

[27] Wang M., Wang Q., Liang K., Han K. A Channel Error Calibration Method for Circular Array Radar Based on Curve Fitting. Proceedings of the 2022 IEEE 5th International Conference on Electronics Technology (ICET).

[28] Li W., Lin J., Wang W., Wang Y., Chen Z. Method of multi-channel calibration for digital array radar. Proceedings of the 2015 European Radar Conference (EuRAD).

[29] Zhou P., Xue L., Zhang Z., Wang Y., Zhang X. (2021). Research on the Parameter Design Method and System Simulation of Multimode Microwave Remote Sensors Operating in Scatterometer Modes. IEEE Access.

[30] Lu Y.E., Yan Y.G., Yuan J.G., Wu D., Dai C., Yuan H. The Effect of Channel Mismatch and Mutual Coupling on GPS Adaptive Antenna Array. Proceedings of the 2006 CIE International Conference on Radar.

[31] Lin C.-C., Stoffelen A., de Kloe J., Wismann V.R., Bartha S., Schulte H.-R. Wind retrieval capability of rotating, range-gated, fanbeam spaceborne scatterometer. Proceedings of the International Symposium on Remote Sensing.

[32] Naderi F., Freilich M., Long D. (1991). Spaceborne radar measurement of wind velocity over the ocean—An overview of the NSCAT scatterometer system. Proc. IEEE.

[33] Lin C.-C., Rommen B. (2000). An analysis of a rotating, range-gated, fanbeam spaceborne scatterometer concept. IEEE Trans. Geosci. Remote Sens..

[34] Ma W., Du Y., Liu G., Yu Y., Yang X., Yang J., Chen K.-S. (2021). Study on direction dependence of the fully polarimetric wind-induced ocean emissivity at L-band using a semi-theoretical approach for Aquarius and SMAP observations. Remote Sens. Environ..

